# Protection of the rights of the individual when using facial recognition technology

**DOI:** 10.1016/j.heliyon.2022.e09086

**Published:** 2022-03-11

**Authors:** Jawahitha Sarabdeen

**Affiliations:** College of Law, Prince Sultan University, Riyadh, Saudi Arabia

**Keywords:** Facial recognition technology, Privacy, Data protection, Civil law, Criminal law and ethics

## Abstract

The Facial Recognition Technology (FRT) is used to match a photo of a person's face through a database that contains picture, name, and other records of someone that are already in the database. This technology uses biometric data with other available information and provides precise and accurate information about a person and his behaviour. FRT has positioned itself significantly advanced among all biometric-based technologies. The use of FRT by government agencies and commercial organisation comes under scrutiny as many of them use the technology in violation of right to privacy where the data subjects are either not informed of data collection or not consented for the data collection, use or storage of their data. Privation of regulatory measures allows government agencies and commercial organisations to operate with no real legal restraint and only under limited self-regulation in many common law countries. The research focuses on suitability of the existing law to regulate the use of FRT by analysing the criminal law and the civil law including the privacy laws in few common law countries. The analysis of the laws shows that passing of appropriate laws is inevitable as the existing laws are inadequate to regulate the use of FRT by government and commercial organisations.

## 1 Introduction

The easy accessibility and increased use of FRT by government agencies and commercial organizations create concerns regarding privacy, transparency, and accountability as there is lack of regulation on FRT (Bacchini et al., 2019) in many common law countries. The FRT allows someone to run a photo of a person's face through a database where the algorithm in the program will match the face with a picture, name, and other records of someone already in the database and public places. Matching is done by calculating the distances between facial features (Lochner, 2013) and a determination is made, either by automation or human verification, as to whether two images constitute a matching (Snyder, 2018).

Government use the FRT for various purposes, notably for law enforcement and such usage is not disclosed, and people are not aware that they are under state surveillance. The use of FRT by law enforcement has been marked by criticism of bias, discrimination, and lack of transparency. For instance, Bacchini et al. (2019) claimed that the technology affects black community disproportionately as black people are overrepresented in many of the law enforcement databases. Their information has been searched and their face have been matched by the technology for a given suspect, and such an identification may include both accurate and inaccurate identification. Since individuals with darker skin colors may be more difficult to contrast with facial features, mistaken identification may happen frequently. Klare et al. (2012) after comparing six different face recognition algorithms found inaccuracy in facial recognition technologies. The inaccuracy among black people is much higher than the other racial communities. As most software programs are produced by Caucasian or East Asian software (Breland, 2017; Phillips et al., 2011), they recognise Caucasian and East Asian more accurately than other ethnic or racial groups. For instance, it was found that the key facial recognition systems from Microsoft, IBM and China's Megvii failed to accurately identify dark-skinned women (Biometricstoday.com, July/August, 2020).

The Big Brother Watch in UK an independent non-profit organization fears that the disproportionate misidentifications will lead to over policing of ethnic minorities (Big Brother Watch, 2018). The FRTdriven bias heightens the black community members’ self-identity more as members of one race than their professional, religious, or cultural membership compared to other community members like Caucasian. Bacchini et al. (2019) noted that face recognition technology has had heavy impact on self-identification of individuals by adversely identifying them as races, created boundaries and racial discrimination.

Lack of regulatory measures allows commercial organisations to operate with no real legal restraint and only under limited self-regulation. The FRT allows the corporations to identify the customers online and monitor their consumer behaviour without the customers knowing that they have been observed. Retailers can upload the photographs of customers and persons of interest into a facial database and notify the retailers when they enter so that retailers can use the information for better customer service or prevention of any crimes in their retail stores. Though it could be argued that the technology allows the retailers to customise services and provide better management of the store, there is a lack of, or no notification of use of FRT or further use of the videos or photograph taken in the store which could be treated as a violation of privacy and could go against the trust the consumers have on the retailers (Brinckrhoff, 2018).

The FRT could also be used by employers to program wellness and disability, hiring, promotion and long-term care in an unfair manner. When the employers get to know certain biometric information of impending illness of potential or current employees before hiring or promotion, the employer may deny the opportunity without disclosing the real reason for the employer's decision. Similarly, insurance companies may use FRT for eligibility as well as determination of premiums (Mohapatra, 2016). The use of FRT will cause so much stress and possibly violate the right to privacy. When sensitive information like biometric is being collected and used for unintended purposes, there is high likelihood where people become cautious and start applying self-censorship, and that will defeat the freedom of speech and expression. Additionally, continuous collection of images and data using facial recognition technologies by government and commercial organisations may reduce accountability for those who use the data to make decisions (Snyder, E., 2018). Like fingerprint and DNA profiles, the FRT creates data and images that are inalterable in nature as such special protection and consideration should be given to protect the data privacy. The recent report by the Canadian Privacy Commissioners clearly vindicated that the Clearview in many instances used FRT to falsely represent images that caused reputational damage and created distress to concerned individuals. In Clearview's operation, the facial images have been collected and used in violation of privacy laws and the Clearview was asked to cease its operation in Canada and delete images and biometric facial arrays collected from individuals in Canada (Burns et al., 2021). Unlike Canada, many common law countries have not adopted a separate data protection or privacy law or amended the exiting civil and criminal laws to address privacy issues that might arise when biometric technologies like FRT are used. As such, the current state of laws in many common law countries is a poor fit for adequately addressing the issue of violation of privacy rights or compensating the victims if they choose to file a civil case against violation of their biometric information or any other personal information.

This research article aims to analyse the adequacy of the existing laws to regulate the use of FRT in selected common law countries. Accordingly, it investigates the applicability of criminal and civil laws with a view to control the violation of privacy, the possibility of the liberal interpretation of judiciary in extending the current law to curb the use of FRT in violation of right to privacy, and to create accountability. The research also intends to show that the users of FRT may follow certain ethical norms based on social contract theory to respect the privacy of the customers, however, the use of FRT is so profitable and easy to use to get fast results, the users may fail in their ethical commitment of their business operation.

The research used legal logical and content analysis. The author analysed the relevant applicable laws and cases that focused on its applicability and the adequacy to regulate the use of FRT in common law countries using logical reasoning. The logical reasoning of exegesis and deduction considers the past court decisions and legislation in arriving at suggestion and conclusion. In this analysis, relevant laws and decided cases from few selected common law countries like the United Kingdom (UK), Canada, Australia and the USA have been taken as examples to highlight the adequacy of laws in regulating the FRT. To achieve the research objective, the researcher analysed the criminal law that may be applicable to regulate the telecommunication devices as the technology used in FRT is related to telecommunication. And it seems appropriate to relate the telecommunication law to regulate FRT to be in parallel with regulation of use of communications data. For instance, Australian Telecommunications (Interception and Access) Act 1979, Telecommunications Act 1997 (Cth) and the Canadian Criminal Code 1985 were analysed to show that the current legislations on criminal law and telecommunication law only have a general prohibition on intercepting, storing, and transferring voice and texts messages that are going through the telecommunications system and there is no protection available for visual images. Based on the analysis, it is possible to show that the current criminal law and telecommunication law of common law countries that have similar provisions/mostly similar provisions could consider amending the law to include visual images in their legislation so that it can be considered as part of *actus rea* in the commission of a crime. The *mens rea* of the criminal action will be based on the intention of the party in accessing visual images and accordingly the respective legislation should be amended. The civil law principles of *unconscionability*, *intrusion upon seclusion, breach of confidence and misappropriation* that are applicable in the UK, Canada. Australia and USA were also analysed. These common law principles that are applicable in civil matters have been used to rectify privacy and data protection violation in the absence of specific data protection law. In case of data protection legislative initiative, the UK Data Protection Act, 2018 was taken as an example since the UK Data Protection Law 2018 is comprehensive and up to date among the data protection legislation in common law countries. UK was part of EU member countries and amended its data protection law in par with EU General Data Protection Regulation 2018. Since USA has no specific legislation in relation to privacy, the fourth amendment to the USA Constitution was analysed to show the possibility of recognition of privacy rights pending federal law on privacy protection. Some states like Illinois passed law to regulate FRT as such discussion of the Illinois Biometric Information Privacy Act (BIPA) 740 ILL. COMP. STAT. 14 (2008) was also added in this research.

In analysing the literature, the content analysis was used to study the theoretical content and applicable legal principles as described by journal articles on the related topic (Srinivasan, N.D). Using this approach, the author analysed relevant literature from popular databases like Scopus, Lexis Nexis, Westlaw, Web of Science, Science Direct, Emerald and Directory of Open Access Journal and provided input into the availability and applicability of the laws under common law to regulate FRT. The researcher also collected news reports of current affairs on the research topic from World Wide Web. Relevant keywords and phrases like “Face recognition technology”, “invasive technology”, “privacy and facial recognition technology”, “data protection and face recognition technology”, “criminal laws and privacy protection”, “civil laws and privacy protection”, “ethics and facial recognition technologies”, “breach of confidence” and “intrusion upon seclusion” in all possible combinations were used to collect relevant literature on the research topic. After collecting available literature, laws and cases, the researcher sorted out the literature on FRT and law in relation to data privacy and mapped 64 relevant literatures besides the legislation and decided cases, as relevant research materials for the analysis of this research. The legislation and cases were selected for its relevancy to the research topic on data privacy or its relevancy in regulating communication technologies in relation to data privacy.

The article is structured as follows: the first section introduces the research topic, the objective of the research and the research methodology. Analysis is presented in section two and a separate section of recommendation is discussed in section three. The research is concluded in section four.

## 2 Analysis

### 2.1 Violation of data privacy in the use of facial recognition technologies

FRT unlike other technologies said to be invasive, can collect unique biometric data of oneself without consent and could match with other available data (Sprokkereef and de Hert 2012). It has the capacity to monitor someone around the clock against the right to be anonymous (Odoherty et al., 2016). At the early stage, this technology was used for limited purposes by the government agencies and law enforcement, and later, many organisations started using it for business purposes (Zhao et al., 2003; Unar et al., 2014). North-Samardzic (2019) citing Sprokkereef and de Hert (2012, p. 82) mentioned that intelligence, distant sensing along with passive biometrics that do not require corporation of data subjects can challenge the data protection rules and regulations. In the use of FRT, there is also a power imbalance where the people may not be able to challenge the data collection and sharing activities of big companies as well as government.

The online footprint is the realty of life, people seek to protect privacy even if they shared their information freely online. Corcoran and Costache (2006) stated that people share the information and images on the internet at the same time they expect some level of privacy and control over shared information or images. Conversely, the commercial organisations collect and share publicly available information without further notice to the owners in violation of privacy. Privacy is considered as a value which gives autonomy for individuals. The value includes both freedom to decide and freedom to deals with own personal information (Hyne, 2021). The privacy could include any sort of communications about one's private life. The Article 12 of the *Universal Declaration of Human Rights* (1948) (Proclaimed by the General Assembly, resolution 217 A (III), A/RES/3/217 A, 10 December 1948) clearly states that there should be no arbitrary or unlawful interference with his privacy, home, or correspondence, nor to unlawful attacks on his honor and reputation.

### 2.2 Use of FRT by government agencies and privacy concerns

Various government agencies including the law enforcement are using the FRT to verify somebody's identity (face verification) or to identify an unknown face (face identification) (Jones, 2021). The technology is said to be useful as it could help in investigation, collecting forensic evidence and identifying victims. The law enforcement data bases, for instance, generally consist of DNA, fingerprints, and picture of a person in lawful custody or previously arrested persons. In a Special Report of the Office of the Privacy Commissioner of Canada (2021), it is stated that the deployment of FRT could support national security objectives with relatively less cost and it can easily be added to the existing surveillance system. Similarly, the white paper from the World Economic Forum in collaboration with the International Criminal Police Organization (INTERPOL), the Centre for Artificial Intelligence and Robotics of the United Nations Interregional Crime and Justice Research Institute (UNICRI) and the Netherlands states that FRT has the potential to help resolve crimes, find missing persons, and prevent crimes. However, there are potential for serious violation of privacy and fundamental freedom if the use of FRT is not regulated appropriately (Madzou et al., 2021).

The concern of privacy violation could be heightened when law enforcement uses FRT and accesses other civilian databases with images of public without their knowledge or consent. In US, for instance, police have access to Mugshot images along with state driver's license databases. Such access allows innocent people's pictures to be matched for law enforcement purpose. FRT trials conducted in Berlin, London, and Cardiff show that FRT produced large false-positive matches (Coleman, 2019; Fussey and Murray, 2019). The possibility of bias may arise as the data used to input may not be explicitly programmed according to various purposes and if the original data has flaw or has bias the output will reflect the same. Relying on false result causes serious consequence to an individual private and public life and freedom.

The FRT is also used by other government agencies for various purposes. Gorman, (2021) citing the Government Accountability Office (GAO) Report stated that the most U.S. executive departments are using FRT. Among the 24 federal agencies and 15 executive departments, the GAO Report found that majority of them have plan to continue using them through 2023. FRT is mainly used for digital access, law enforcement, and physical security. The Departments of Homeland Security (DHS), Defense, and Justice employed two-thirds of federal facial recognition systems. The government also builds their own databases and use commercial software to conduct facial recognition searches. Defense Department, DHS, Immigration and Customs Enforcement (ICE) and the Secret Service used the controversial Clearview AI's FRT. Clearview is alleged to have collected 3 billion images by scraping personal images from social media profiles. Additionally, ten agencies engaged in research and development of FRT systems, and four other agencies entered into FRT-related agreements with non-federal partners, and they even hosted Biometric Technology Rallies for facial identification of people wearing masks. As the GAO report demonstrates, federal departments are poised to expand their use of facial recognition systems across a wide range of cases in the absence of federal regulation (Gorman, 2021).

The situation is not better in many common law countries where there is no clear legislation on regulating the use of FRT, the law enforcement operates with minimum accountability and transparency. It seems like with the government surveillances, citizens' freedom is being determined based more on their color, race and neighborhood they live than any other criteria (Jones, 2021). Due to the potential for inaccuracy, bias and discrimination in the use of FRT, many countries in Europe and few cities in US such as San Francisco, Oakland and Boston have banned the use of FRT by public agencies, while the other states have introduced legislation to regulate its use. At a more global level, the United Nations Office of the High Commissioner for Human Rights’(OHCHR) recent report on the right to privacy in the digital age recommends government halt the use of remote biometric recognition in public spaces in real-time until they can show there are no significant issues with accuracy or discriminatory effects. It also suggests that these AI systems must comply with robust privacy and data protection standards (OHCHR, 2021). Lewis (2021) suggests that regulating the government use of FRT should parallel by the regulation of use of communications data. The prior approval for programs, rights against unreasonable searches, requirement of search warrant, transparency in use, rules limiting secondary uses of collected data, and redress mechanism and strict oversight of the use should be similar to the collection and use of communications data. Government uses of FRT other than law enforcement and commercial use may include consent, transparency and oversight, acceptable secondary uses, and redress mechanism. Introduction of new regulation or amending the existing laws is inevitable since the communications and criminal laws failed to regulate the use of FRT and may not be useful to prosecute the abuse of technology.

### 2.3 Use of FRT by commercial organisations and privacy concerns

The world business operations and the "Golden Age of Travel," prior to pandemic came to a halt with the rapid growth of the pandemic that affected the tourism and other businesses to an unbelievable extent. According to the UNWTO World Tourism Barometer released in July 2020, there was a drop of 98% fall in international tourist numbers in May when compared to 2019 (UNWTO, 2020). Ivasciuc, S.I. (2020) citing United Nations (2020) states that recovery of tourism and related business will depend on taking advantage of technology to better understand, monitor travellers’ needs, trends, and use digital platforms to enhance the competitiveness, satisfaction, and agility.

In this context, FRT becomes one of the important technologies that has the potential to help in recovery of many sectors after pandemic. Ivasciue (2020) states that FRT could be used for security and access purposes. It could be used to grant guests access to their hotel room and to identify problematic guests who had a history with the hotel chain. It also could be used for enhancing customer service. Employment of FRT will allow identify guests and deliver more personalised greetings and a more tailored service. Additionally, Payment Authorisation could also be done promptly and efficiently like how MasterCard uses. MasterCard uses FRT for customer verification and a customer can confirm a payment using the camera on their phone, or a camera supplied by the vendor (Ivasciuc, S.I. (2020)).

Many other sectors like airlines, retails, healthcare, and accommodation rental services are using FRT to expedite boarding, to eliminate the need for residents to have identity, speed up registration and inquiry processes, and to provide better services (Klein, 2019). The FRT in healthcare helps to identify, monitor patients, diagnose genetic, medical, and behavioral conditions. Algorithms or machine learning are used to find patterns in facial features for verification or authentication purposes, diagnosing genetic disorders and providing accurate health care (Martinez-Martin, 2019). FRT could also be used to assist in earlier detection and treatment of genetic disorders along with the prediction of behavior, pain, and emotions by identifying facial expressions. With the good quality data input and analysis, privacy and data protection measures, concern of violation of privacy could be minimised and the FRT could be used to benefit the customers as well as the businesses.

In this regard, it can be said that in order to benefit from the use of FRT, it should be ensured that privacy and data protection are given enough consideration. Privacy is an individual's life condition that excludes inclusion from publicness. Privacy includes data privacy too. In the context of FRT and other computing systems, the data privacy is pertinent as it allows control over personal data of individual. Privacy in general is considered as part of individual identity, as such in Europe, individual privacy is conceived as a fundamental human right and government provide laws to regulate any invasive activities following the Universal Declaration of Human Rights (1948). In common law countries, only a few countries like UK and Canada enacted laws to adopt the Declaration and protect privacy, while other countries protect privacy with the existing laws and regulation. However, there is a growing demand internationally to regulate technologies like FRT that uses biometric data (Martinez-Martin, 2019; Selvadurai, 2015; Jones, 2021). There are suggestions to introduce privacy or data protection laws akin to that of the European General Data Protection Regulation (GDPR) with some modifications or additions to cover AI, FRT and other related technological developments (Jones, 2021; Gahntz, 2020; Office of the Privacy Commissioner of Canada, 2021).

### 2.4 Regulating the FRT through criminal law

The introduction of criminal liability for unauthorized application of FRT to online visual images is unavoidably inevitable as the use goes against the public interest. The current law in many common law countries is inadequate to regulate the use of FRT. Some countries like Australia through the telecommunications interception and access laws may try to regulate the use of FRT. However, the telecommunications interceptions and access law only protect voice data and text, not visual images. The Chapters 3 and 4 of the Australian Telecommunications (Interception and Access) Act, 1979 make it as an offence on anyone who intercepts live communications passing over the telecommunications system or store the communication data and allow others to access the data without the knowledge of neither of the intended recipient of the stored communication nor the person who sent the stored communication. Section 13 of the Telecommunications Act, 1997 (Cth) also criminalises the disclosure and use of telecommunication information by service providers. Though there is a general prohibition on intercepting, storing, and transferring voice and texts messages that are going through the telecommunications system, there is no protection available for visual images.

Canada has amended its Criminal Code, 1985 to prohibit transmission of intimate images online. The offence under this section is considered as a relatively new offence that was created in response to growing concerns about violations of privacy and applied to control cyber-bullying, distribution of intimate images in a public forum without the consent of the owners of the images. Since such a distribution created emotional hardship and shame to the victims, the court considered it as serious crime. Section 162 (1) of the Criminal Code further provides that “no person shall be convicted of an offence under this section if the conduct that forms the subject-matter of the charge serves the public good and does not extend beyond what serves the public good. The motives of an accused are irrelevant”. However, if the distribution of the images is justifiable under public good, the person distributing the images is not criminally liable. The main objectives of penalising these activities are said to be denunciation and general deterrence (*R.* v*. Calpito*, [2017] O.J. No. 1171 (O.C.J.)).

In *R*. v*. Dewan (*2014, O.J. No. 5151 (C.A.), the court applying s.162 (1) concluded that publication of an intimate image without consent was a serious offence. The offender distributed an intimate email purportedly from the victim to nine colleagues. In *R.* v*. B.H*. (2016, O.J. No. 7080 (O.C.J.), the Ontario provincial court was dealing with an offender who was a university graduate posted videos to a porn website supposedly. They were encrypted and required a password, the accused had sent the relevant website link and password to numerous friends. The court found him guilty of distributing objectionable images without consent. The amendment was used to control the deliberate and vindictive publication as it had a serious, profound impact on the victims (*R*. v*. J.B* 2018, O.J. No. 4133). Nonetheless, this new section may not be able to help if the images are not intimates in nature and they have been used for commercial and law enforcement purposes. The only alternative for the Canadian is to rely on the privacy laws.

The use of FRT with other advanced technologies is known to undermine privacy that potentially may lead to other crimes like harassment. The violation of privacy in FRT occurs when facial features and appearance identify an unknown party from a set of known possibilities (Selvadurai, 2015). The in-depth information gathered through social media sites with publicly available images could create quite an intrusive profile of any individuals. Likewise, retailers who have collected data on customer preferences and demographics over the course of business may opt to use FRT to understand consumer behaviours and preferences in a real-time basis. This has increased the likelihood of consumer privacy violation.

According to Selvadurai (2015) privacy is public interest matter, and on this basis of public interest, the criminal law could prohibit the unauthorized use of face recognition software. In criminalising the unauthorized application of FRT to visual images on the internet, it should prohibit an image that is disseminated in one platform to be retransmitted in another context or platform without the consent of the owner of image or without lawful justification. It should also provide that an image that is disseminated in public does not vitiate the consent requirement for the further uses or to the application of biometric facial recognition technology. In this context, the case of *Campbell* v*. Mirror Group Newspapers Limited*, ([1998] 2 SCR 591) could be relevant and the rationale of this case can be applied to the use of image collected online using FRT. The House of Lords in this case stated that where a photograph of a well-known model leaving a rehabilitation clinic in a public space was published without the model's consent and thus violate duty to confidentiality.

The recent Canadian decision of Privacy Commissioners on Clearview AI supports this approach as it indicated that images available in public domain does not mean that privacy right attached to it has been abrogated simply because the images and contents were available in social media or in public domain. The people who uploaded the images are still in control of them and will have to give consent for any use of technology application of those images or the usage should come within legal derogation of law (Burns et al., 2021). In this case, the use of a facial recognition tool by Clearview AI, Inc. was investigated by the Privacy Commissioners of Alberta, British Columbia and Quebec and their federal counterpart in Canada. The Privacy Commissioners jointly found that Clearview's treatment violated provincial and federal private sector privacy laws and recommended that Clearview cease all operations in Canada and delete all images and biometric facial arrays about Canadians in its possession. The finding shows that:

1. biometric information is inherently sensitive.
2. the privacy legislation has extra-territorial application, and
3. information that is available on the internet is not necessarily public and will need to satisfy consent requirements under privacy legislation (Burns et al., 2021).

Similarly, in *Aubry* v. *E′ ditions Vice-Versa Inc* [1998] 2 SCR 591, the Supreme Court of Canada held that there had been a violation of a young woman's right of privacy under Article 5 of the Quebec Charter of Human Rights and Freedoms when she had been sitting on the steps of a public building. In the case of *Cadillac Fairview*, the company was instructed by the Canadian Federal Privacy Commissioner to obtain express consent to collect, use and discloses information when they use facial recognition technology (Office of the Privacy Commissioner of Canada, 2020). These can support the proposition that publicly available information and images do not lose privacy protection. The use of those information and images by others through technology like FRT should be treated as violation of privacy and there is no inference of implied consent attaching to the use of the image without the consent of the concerned individuals or legal justification.

### 2.5 Regulation of FRT through the civil law of tort

Many social media companies use the FRT in a robust and advanced manner to recognise the faces with high accuracy. Facebook, for instance, make the users agreed to the broad privacy policy that allows Facebook to use the images freely, such use will go against the initial intended consent of use in the contract. In the absence of appropriate privacy law to regulate the use of publicly available images, the existing civil laws may be applied in limited scope. The doctrine of unconscionability could be used to control the use of images without consent if the parties have contractual relationship. To claim under unconscionability of contract, it should be shown that there is a need to show substantial degree of unfairness beyond a bad bargain (*Sonic-Calabasas A, Inc.* v. *Moreno*) (Cal. 4th 1109, 1160 (Cal. 2013). The court may treat a contract unconscionability if it is against public policy (*Szetela* v. *Discover Bank*) 97 Cal. App. 4th 1094, 1101 (Cal. App. 4th 2002) or there is a presence of ambiguous phrases like “as long as” and “for a reasonable period” that allows the companies to misuse data and images without limitation for a considerable amount of period. Since unrestrained use of data including biometric data by the companies put consumer interests at jeopardy, the courts may be opened to consider the issue of unconscionability (*Spokeo, Inc*. v. *Robins*, 136 S. Ct. 1540, 1547 (2016)). To apply the doctrine of unconscionability in contract, it will be required to show that:

1. There is an absence of meaningful choice, and the usage is unfair to one of the parties in the contract.
2. There is an unequal bargaining power, and
3. The contract is harsh on the side (*Williams* v*. Walker-Thomas Furniture Co.*, 350 F.2d 445, 449 (D.C. Cir. 1965)).

When the customers enter a typical standard form of contract, element of oppression could happen due to inequality in bargaining power where there is no other choice is available for one of the parties. For instance, the users will be left out from a popular social media if they do not opt in for the terms of conditions of Facebook. The inequality of bargaining power to the contract was illustrated in the US case of *Ting* v. *AT&T* (319 F.3d 1126, 1133–34 (9th Cir. 2003). AT&T mass mailed a Consumer Services Agreement about a binding arbitration clause to customers, the court declared the arbitration clause as invalid. This case can be applied to any companies that are using standard form of contracts, if it is shown that:

1. there was no meaningful opportunity to negotiate with the company when agreeing to the Terms of Service or Data Policy,
2. the contract was entered into as strictly on a take-it-or-leave-it basis. The lack of choices in opting out from facial technology usage and the lack of information about company's retention and deletion of biometric data could imply that the parties are not given opportunity to negotiate. Some companies even failed to mention their use of facial recognition technology to collect and analyse data. Therefore, agreement to the terms and conditions of the usage of the services should not be interpreted to allow the use of FRT since there is no agreement on the usage of FRT. For instance, Facebook never disclosed its usage of FRT to its users, and they are not aware of Facebook usage of FRT when they agreed to the terms and conditions (Brinckrhoff, 2018). Like Facebook, most of the companies do not disclose the use of FRT and exempt themselves from liabilities and therefore the consumers do not know what is being collected, used and stored about themselves (Brinckrhoff, 2018).

Intrusion Upon Seclusion may also be used to stop the use of biometric data through FRT and to claim compensation for the wrong done to individual's reputation. It is a tort claim that can possibly be used by the customers or users in case of privacy violations. In the Canadian case of *Jones* v. *Tsige* (2012, ONCA 32), the court awarded the plaintiff damages against defendant who accessed her financial information without consent. In order to bring action under this tort it is necessary to establish the following elements:

1. The defendant's conduct is intentional, or reckless.
2. The action invaded plaintiff's private affairs or concerns without any lawful justification.
3. A reasonable person would regard the invasion as highly offensive causing distress, humiliation, or anguish (*LAC Minerals Ltd* v. *International Corona Resources Ltd*, [1989] 2 SCR 574).

If any one of the elements is missing, then the action cannot be sustained. The tort of intrusion upon seclusion was also considered in the recent Superior Court of Ontario case of *Agnew-Americano* v*. Equifax Canada Co*. ([2018] O.J. No. 361) where the court affirmed the possibility of bringing action under intrusion upon seclusion if personal privacy which protects the right to bodily integrity or territorial privacy which protects personal space that is necessary for reasonable expectation of privacy or information privacy that protects information about ourselves is violated. Intrusion into any types of privacy could be considered as actionable. In FRT case, any photographs or images that have the tendency of affecting bodily integrity and exposing activities to others whom the owner of the images does not want to share could be viewed as highly offensive to a reasonable person and that caused distress, humiliation, or anguish to an individual.

There is also possibility of filing a civil action under breach of confidence. This tort could be applied to protect the unwanted disclosure of private information if confidential information is entrusted to a person for a limited purpose, there was a disclosure, and the disclosure caused injury. This remedy can be extended to third parties if they receive information from an individual who is under an equitable duty of confidence (Hunt 2012). The House of Lords in *Campbell* v*. MGN* (2004 UKHL 22), dispensed with the requirement of entrusting confidential information and disclosure. Accordingly, an action can be brought against a stranger if the private information is accessed and disclosed.

The English Court of Appeal in *Tchenguiz* v. *Imerman* (2010 EWCA Civ 908) followed *Campbell*'s case and decided that the defendant will be liable for breach of confidence if he accessed private information even if he did not disclose the information. The Canadian courts too accepted the traditional principles of breach of confidentiality. The Superior Court of Justice reiterated that if the elements of breach of confidence are proven, it is possible to succeed under this tort for violating one's privacy (*Doe 464533* v*. N.D*., 2016 ONSC 541). The problem in relying on breach of confidence is that the information must be parted in confidence and the defendant must have accepted the information in confidence. Traditionally, this tort would not extend to stranger who is not in a relationship of confidence with the claimant, or who does not obtain information from someone who was in such a relationship.

Misappropriation in relation to commercial valuable name may also be applied to control privacy violation. However, it will have limited application as it can only be applied for stopping use of an individual's image when the distribution causes damage to his reputation or personal image. The application of misappropriation torts is beneficial for commercial exploitation of individual's name as a brand not for protection of ordinary persons' reputation. Many photos may be taken in public spaces, thought to be private moments, become parts of public lives where the photos are appeared with other information. The court may make an extension of misappropriation as the usage of facial recognition technology not only cause emotional harm to the famous but also to the ordinary people since the display of individuals' faces attract unwanted attention and therefore causes stress. However, the risk is that the court may not favour the restriction of constitutional free dissemination of ideas, images, and newsworthy matter over protection of private information (Harvard Law Review, 2007).

It seems clear that the existing criminal law and the civil law (contract or torts law) have limited application to regulate the use of FRT that violates privacy rights. The only effective way to regulate the use of FRT is to amend the existing laws or create new legislation to prohibit the use of FRT without the consent of the users or legal justification. In the meantime, the judiciary's role in liberal interpretation of law in case of publicly available information is considered crucial so that information will not loose privacy protection simply because the images were available in public places.

## 3 Recommendations

### 3.1 The amendment to criminal and civil laws

As the current legislation (telecommunication and criminal law) failed to address the technology induced crimes and breaches, it is necessary to criminalise the unauthorised use, storage and communication of images through FRT. The amendment to laws in most of the common law countries should reflect the novel nature of FRT and the related technologies while considering the very action of uploading of the image of the non-consenting individuals and/or the identity of the individuals as *actus rea* of the criminal action. Similarly, any storage of digital visual images or communications should also be considered as part of *actus rea*. The prohibition to the application of facial recognition technologies should be treated same as the existing prohibition of interception of telecommunication transactional data. In formulating the laws, consideration should also be given to recognise the creation of private spaces within public space of the Internet. There could be boundaries for each protected space as the same way as unauthorized access of material stored on telecommunications systems (Lipton, 2009–2010).

The *mens rea* of the criminal action should be based on the intention of the party in accessing visual images through the unauthorized application of FRT. Any proposed amendment or new law in common law countries like the UK, Canada and Australia should include exception like national security, law enforcement or other legitimate purposes like economic well being. The exceptions should be clearly demarcated so that abuse by law enforcement agencies can be controlled (Selvadurai, 2015).

In cases of application of common law principles in civil matters including privacy violation, some common law principles are utilised as there is no specific law to control violation of privacy in many common law countries. However, the extent of protection provided by those principles is not clear. For instance, in applying intrusion upon seclusion, it is not certain if the court is willing to apply intrusion upon seclusion for a matter happened in public places. The few reported cases on public inversion of privacy are involved celebrities or business personalities. The court may take different approach in case of ordinary individuals’ invasion of privacy in public places even if the activities have intruded into a private place or conversation (Harvard Law Review, 2007). Furthermore, the courts may be careful not to limit free flow of information as such they may limit the privacy protection in public places.

Similarly, when the tort law principle of breach of confidentiality is applied to remedy the harm caused by disclosure of confidential information, its application will be very limited. Breach of confidence could only protect parties directly involved, who received the information in confidence. If the courts follow *Campbell* and *Tchenguiz* decisions as discussed earlier, it may be possible to apply broadly on whoever accessed and disclosed the information without the need to prove an existing confidential relationship. However, if the courts refuse to apply this tort to strangers, breach of confidence could only be applied to corporations or government entities who have collected data previously for business or other related purposes (Hunt, 2012).

Misrepresentation of commercial valuable name is another principle that have been used to protect individual from intrusion as it may cause emotional harm. The current application of this principle only protects the famous while the ordinary people are excluded from the protection of this principle. It is not certain if the court is willing to use this principle often as it may go against the free speech and newsworthy matter (Harvard Law Review, 2007).

Perhaps, legislative intervention in many common law counties including Canada, Australia and the UK clarifying the application of FRT will give a win-win situation for individual, the regulators, and the industries as the legislation could clarify on the requirements in using the FRT. The legislation could also be made clear possible is consent requirement or any legitimate justification to the requirement of consent. As regard to consent, it could make it clear that the consent obtained should be express consent and there should not be any inference of implied consent to the use of the images obtained by FRT in normal circumstances. Derogation to general principles, however, could be added to the requirement of consent.

### 3.2 Introduction of data protection law to regulate the use of FRT

Data protection law in GDPR style like UK Data Protection Act, 2018 may control invasions of privacy in the use of FRT. Under the proposed data protection law in regulating the FRT, the collection of personal data of individuals like biometric data should only be collected after obtaining express consent. The UK Data Protection Act, 2018 along the line of GDPR states that biometric and health data are sensitive data and collection or processing of these data should only be done after obtaining express consent from concerned individuals or the collection should come within exceptions mentioned under the law. Sensitive data includes biomatrices data and data related to an individual's religion, political opinions, sexual orientation, and genetic data (Article 9, GDPR and Section 10 of the UK Data Protection Act, 2018). Just like the UK Data Protection Law, 2018, express consent is required unless the collection and use of sensitive data are for legitimate use. For example, the use of FRT in health care generally requires express consent for collecting and storing patients' images and for using the images for other related purposes. Even if data may be anonymized, there might be a possibility to link back to the original owner of the data, thus getting consent will be advisable and it will also create better image of the organisation which uses the FRT. Any law in this aspect should also prohibit collection of personal information from a third party who collects personal data unlawfully. Collecting data from companies like Clearview AI which uses FRT without consent should not be allowed. There should also be prohibition of state surveillance cameras to be installed only in areas where minorities live, and the database comprising wanted or convicted people in Mugshot should not include convicts of minor misdemeanors to avoid over representation of minority in the Mugshot.

In term of accuracy, there should be requirement that the decisions made about individuals should not rely solely on FRT and there should be a requirement of human intervention before any action is being taken. Certain benchmarks on facial recognition systems should be devised and the use of FRT or similar technology should produce same or less errors as human judgements, otherwise the use of FRT should not be allowed. It should also require a similar error rate across different sub-populations with same gender or skin color. These measures to ensure accuracy could help to balance interest of various stakeholders. The data protection law should also include the principles of necessity and proportionality. This will ensure that privacy-invasive practices are carried out for a sufficiently important objective, and that they are appropriately tailored so as not to intrude on privacy rights more than necessary. The law enforcement should show that the use of FRT is more than mere “usefulness” to meet the necessity and proportionality principles. There must be evidence of pressing and specific reason to use the technology and it may not be enough to rely on mere public safety requirement (Office of the Privacy Commissioner of Canada, 2021). The requirement of data minimization should be included so that there should be measures to collect what is necessary and reduce the risk of over-broad data collection. The users should be accountable as to what is being collected, how it is being collected, who collected the data and the purpose of collection along with safeguard taken to protect the collected data.

Transparency requirement should also be added to any legislation. Transparency means disclosing the collection and purpose of collection so that one can understand the use and process of his data and can decide if these purposes are acceptable to him (Aden, 2022). Section 35 of the UK Data Protection Act, 2018 like Article 5(1) of GDPR incorporated transparency as one of the core principles for data protection. Transparency is part of fairness principle. However, those government and commercial organizations trying to attain asymmetric power through data, try to ignore transparency and fairness (Aden, 2022). To afford individual's rights and to balance the business and public safety requirement, it becomes apparent that the government agencies and commercial organisations should disclose the ways they are using FRT, the places where images are taken, how the images are used. Such notice to the public regarding FRT use or intention to use FRT allows for public input and in turn could create trust between the possible data subjects and data users. This initiative will allow the individuals to change their behaviour in public if the data subjects wish to do so and will also allow the government agencies and commercial organisations to act responsibly where they will be careful in collecting, using, and storing personal data through FRT or other technologies. Furthermore, it could help to avoid bias.

Data protection laws should also prohibit the use of false or outdated personal data and include various rights to data subjects. The UK Data Protection Act, 2018 like GDPR in Chapter 3 provides eight rights to data subjects including the right to be informed, the right to access to data and the right to have the data rectified. The up-to-date data should be used, only after ensuring privacy rights are taken care of so that it could create synergies between privacy protection and justifiable use of sensitive data. The use of FRT should be subject to judicial or regulatory authorization and there should also be introduction of measures to control manipulation that can cause irrevocable harm to individual's physical or psychological behaviour. As privacy gives individuals freedom to live and develop independently as persons away from the watchful eye, only a justifiable intrusion should be allowed. Judicial or regulatory authorization could ensure justifiable intrusion on privacy. The province of Quebec in Canada, for example, has issued a new Guidelines under the Quebec Data Protection Law (Act respecting the Protection of Personal Information in the Private Sector, CQLR c P-39.1) addressing biometrics issues including FRT. The Guidelines requires organizations to notify the provincial Privacy Commissioner before introducing or implementing a biometrics database. The regulator in Quebec may allow or prohibit or order the destruction of the database (Office of the Privacy Commissioner of Canada, 2021). Furthermore. Adding the requirement of warrant for long-term surveillance will control the misuse of FRT by law enforcement and before such warrant could be issued, the government agencies should justify that other means of surveillance have already failed, are likely to fail and the use of FRT is the last resort (Jones, 2021). The requirement for approval may also be added and this requirement should state that the data users should get approval before the use of FRT and the approval request shall include the types of analyses that they are planning to carry out using data generated by FRT, the measures taken to ensure accuracy and avoid bias, if bias is detected, what type of explanation and the rationale for such bias. In term of improving the trust with the use of FRT, training the users should also be added. To control biases of FRT systems, the legislation should require that the data set for the use of FRT should include people of different background, color, and gender (Martinez-Martin, 2019).

In the context of regulatory oversight or authorization, Gahntz (2020) suggested a multi-stakeholder regulatory framework to address concerns about FRT usage. The multi-stakeholder committee should allow representation from industry, government, academia, and civil society. Applications submitted for approval should furnish specific information of FRT use, possibility or probability of errors, possible violation of privacy or other related rights and the redress mechanism. The committee should deliberate the use and the effect of use and decide on majority decision. A public register could be created about the commercial organisations or government departments which use the FRT, and information related to usage. Such a public register would help public to know which of the state agencies and organisations are using FRT. It also gives knowledge of acceptable usage while ensuring transparency. The multi-stakeholder approach could be a solution to address various concerned parties’ interest in FRT.

The legislative measure should include the requirement of security and administrative measure for the repositories of FRT data as it will be a special target for malicious actors, therefore the security measures taken shall ensure protection against external and internal misuse of data. Selvadurai (2015), on the need to enact new laws, mentioned that the new regulation should acknowledge the widespread and consensual sharing of personal information and facilitate the self-disclosure and self-identity while maintaining the control of information shared online. Since privacy allows the individuals to disclose and withhold information about themselves, the principle of selective disclosure and withholding should be allowed without eroding protection of privacy in the digital era. It should introduce granting of selective anonymity rather than control over individual information where the individuals can disclose to someone while denying access to some others of their information as part of control of information (Welinder, 2012). In US, advocates are calling for regulatory measures on FRT as it was done to regulate wiretapping an electronic device through Wiretap Act (18 U.S. Code, 2511) (Ringrose, 2019). It is expected that the new law should regulate FRT and other technologies regarding non-consensual data collection, retention, data error and impose administrative requirement for trained personnel to interpret the technology output (Ringrose, 2019).

As biometric facial data is unique to each individual and does not vary significantly over the period, it is therefore, considered powerful intelligence and tracking tool for business and government use. The introduction of the data protection mechanism could be adopted by some common law countries that do not have specific legislation on data privacy or the countries like Canada whose existing law on data protection is in need of an overhaul. There were many attempts, for instance in USA, made at the federal level, to restrict the use of FRT on private sector use and use by law enforcement. The Facial Recognition and Biometric Technology Moratorium Act, 2021 was introduced to pause the use of FRT by federal agencies or officials without permission by Congress. However, the law is still pending in the Committee level. While the federal law is still pending, some of the states have passed legislation to control or prohibit the use of FRT and allow action for breach of privacy on biometric data (Yahoo.com, 2020; Congress.gov, 2020). Recently, a class action lawsuits filed against Home Depot and Lowe's for using FRT for loss prevention in violation of the state's Illinois Biometric Information Privacy Act (BIPA) 740 ILL. COMP. STAT. 14 (2008). In *Rosenbach* v. *Six Flags Entertainment Corp.* 2019 IL 123186, the Illinois Supreme Court applying the BIPA decided that there is no need to prove injury in biometric data violation, the plaintiff only needs to establish technical violations of the law to pursue a claim and in this case an amusement park was sued by the parent of an underage child for scanning and storing fingerprint without parental consent. Under the current state legislation filling a case for breach of privacy is simplified as the plaintiff only need to show the use of FRT or technologies without consent. States who implemented FRT regulating laws argue that the once biometric data is something which is very personal and the abuse of it could be difficult to rectify it.

In US, in the absence of federal legislation, many hope that the judiciary will expend *Katz*'s (*Katz* v. *United States*, 389 U.S. 347 (1967) principle for the protection of privacy in utilising the FRT and other technologies that use biometric data. In *Katz*, the court stated the need for satisfying twofold requirements to protect the private information or images: an individual should have exhibited an actual (subjective) expectation of privacy and the society is prepared to recognize the (objective) expectation as ‘reasonable’ (389 U.S. 347 (1967). The requirement of “subjectivity” will look into the owners' reasonable expectation of privacy and the “objectivity” will see if the society's preparedness to accept the individual expectation of privacy concerning FRT as reasonable. Since information and images were collected for a specific purpose then the objective expectation of privacy is that it should only be used for that purpose unless the concerned individual consented for further use. Applying Katz's decision to facial recognition technologies, it could be argued that there should be a reasonable expectation of privacy when someone or an organisation collect indeterminate information from millions of people. The society will agree with an aggrieved individual that the collection of information and images should be reasonably free from intrusion (Bacchini et al., 2019). Since the privacy protection in Forth Amendment of US Constitution in *Katz* protects people, not places, the type of technology should not be any hindrance in extending *Katz* decision to FRT and beyond. If *Katz*'s decision is extended to regulate FRT, the government use of a citizen's face without warning and the corporate use of online images for commercial purposes could possibly violate the reasonable expectation of privacy and autonomy of an individual in US.

In this aspect, the decision of *Dow Chemical Co.* v*. United States (*476 U.S. 227, 239 (1986) could be relevant too. The court in this case was of the opinion that photographs taken revealing intimate details may be considered as “search” as such appropriate law should be followed. Similarly, in *United States* v*. Jones* (132 S. Ct. 945 (2012) (No. io- 1259) the Supreme Court of US decided that using Global Positioning System (GPS) device on someone's car and tracking the vehicle on public streets for 28 days was considered as search that requires warrant. If one cannot succeed under *Katz* principle, the other possibility is to adopt a "mosaic theory" suggested by the court in *Jones* where the court may assess actions over a period to evaluate the activities' legality and the possible breach of expectation of privacy. Since the application of mosaic theory and the Kartz's principles are not tested yet, legislators are probably best equipped to protect the public's privacy by enacting appropriate laws to regulate the use of FRT (Lochner, 2013). The cases mentioned show the possible challenges one might face in bringing action in US against breach of privacy by the use of FRT. The available option for the time being is the use of Forth Amendment of US Constitution to protect privacy in the absence of federal legislation on regulating data privacy. The liberal interpretation of the Forth Amendment of US Constitution by courts could be a temporary relief pending federal legislation.

Though the European Union countries have comprehensive data protection law, some countries like France implemented FRT and thus civil activists and organizations like the Electronic Frontier Foundation (EFF) call for the ban on government use of FRT as it could create racial disparities and affect freedom (Ban Facial Recognition Europe, 2020). The German government is in favor of ruling out biometric recognition in public spaces and it supports for an EU-wide ban on biometric mass surveillance and right to encryption of personal communication (Hersey, 2021). The European Data Protection Supervisor (EDPS) asserts that the exemption to allow to use biometrics technology could imply mass surveillance and could go against transparency principle. Recently, the European Parliament adopted a non-binding resolution for a ban on police use of FRT in public places unless it is for fighting serious crimes like terrorism or kidnapping. It also bans the use of FRT on predictive policing where AI is used for profiling potential criminals before a crime is being committed, and private facial recognition databases used by company like Clearview AI. It supported an AI bill to ban social scoring systems which rate citizens' trustworthiness based on their behavior (Heikkila, 2021). Whether the call for ban on FRT use is achievable is yet to be seen as there are countries and commercial organisations using FRT for public health, safety, security, and other justifiable purposes.

In the UK, the Data Protection Law, 2018 and common law could come handy in providing protection for online information and to control the use of FRT in violation of privacy. In *D* v. *L* [2004] EMLR 1 [23], the court stated that it may restrain the publication of an improperly obtained photograph even if the information revealed by the photograph was in the public domain. Though the case is not about the FRT, it seems that the UK courts may be opened to decide against the use of online images for facial recognition purposes (Selvadurai, 2015). In a recent 2020 decision of Court of Appeal, the court decided that the use of FRT should be without violating the rights of individuals. In this case, Ed Bridges, a civil liberty activist filed a case against the South Wales Police for causing “distress” by scanning his face in 2017 and in 2018. The Court of Appeal agreed with him and held the activity of the police breached the right to equality. The case is not relevant to violation of privacy directly, however, the case shows the willingness of courts in UK to apply the existing law to check and control the invasive practices of corporations and law enforcement in the use of FRT (Kirka, 2020). It is to be noted that though the UK cases are not directly binding on the common law countries judiciary, those cases could be considered as persuasive, and it is left to the discretion of the respective common law countries’ court to consider if they want to follow the principles applied in the above-mentioned cases.

## 4 Application of ethics in the regulation of facial recognition technology

Until the appropriate amendment to the legislation is made, the data subjects and users of FRT need to rely on ethics and self regulation. In the context of ethics and self-regulation, the success of all social media depends on complex set of data that are being collected, tracked, and monitored so that users' behaviors and actions could be tracked and predicted (Montgomery, 2015). In case of law enforcement, the suspects or any potential violators of law could be monitored with the use of technology like FRT. The practise is so productive especially in case of corporations and they tried to justify or change their policies to help them maintain the activities rather than abandoning it even if the use of FRT is challenged. For example, Facebook's “Sponsored Stories” for collecting teenagers status updates, check-ins, and “likes” and using the information for marketing to friends has been challenged by the parents. Even though Facebook agreed for a multi-million-dollar pay out plan, it rather introduced changes to data use policy than abandoning the practice (Nick and Joseph, 2014). In the absence of a comprehensive law, the push for industry accountability and corporate responsibility needs to be highly valued (Cohen, 2008).

It is argued that the corporation based on social contracts should meet the expectation of privacy of their customers (Martin, 2016). Since the social contract theory regarding privacy implies that the owner of the information has control over about what, why, and to whom information is shared within specific relationships, the corporations are expected to adequately manage the privacy expectations of the customers. Under social contract, groups, teams, and organizations develop privacy norms to their community dealings as social contract and this creation of social contract allow the parties in the group to negotiate terms and conditions of the use and the resolution of disputes (Dunfee 2006). The forming of group could be related to business or non-business activities.

The social contracts will abide by procedural norm of consent to be part of the community. When information is shared with others the rules will dictate on how to deal with the information received and who else will receive the information (Hartzog 2011). Since social contract is unwritten contract among members within a context or relationship, it is imperative for all members to understand the privacy rule and respect them (Eastlick et al., 2006; Martine 2016). Based on this understanding and respect, the members within a given community share information. Social contract mainly focuses on the responsibilities of the organisations as a contractor to maintain a mutually beneficial and sustainable solution (Nissenbaum 2004, 2009; Martin 2012). In social contract, community and contextual integrity is important. Nissenbaum (2009) explains that protecting the boundaries is necessary while not imposing substantive norms as it may interfere with the autonomy of decision-making regarding privacy. The social contract consists of a locally negotiated contracts with procedural universal principles (Van Oosterhout et al., 2006), the rights of consent, the opportunity to voice their wish and views, and the chance to exit from the social contract (Heugens et al., 2006).

Violation of the norms can occur when the procedural and structural requirements for a legitimate social contract were not adequately addressed and followed by the members. In order to effect the changes to the procedure and requirements, the members are informed through notices. Though the social contract sets the norms and procedure of what, why and to whom the information is shared, the data subjects may not share all the information and wish to keep certain information private and confidential. Under the social contract when information is provided for health purpose, it should be used only for that purpose. If the health provider shares or sells the information for others for marketing or business purpose, the health provider is in breach of social contract. Since the using of the data or images for other than original purpose, sharing with other or selling the data is so lucrative, the organisations are leaned toward violating the social contract that they entered into with their customers.

The researchers in the past (Warren and Brandeis 1890; Persson and Hansson 2003; Westin 1967; Alder et al., 2007) suggested that once private information was shared, there was no reasonable expectation of privacy. However, the researchers at the later period, applying the social contract theory, tried to argue that sharing of private information should not be considered as losing control over data and images and therefore, the expectation of privacy should not be lost. As the owners still have control over the shared information, they should be used in a mutually beneficial and sustainable way as part of social agreements (Culnan and Bies 2003 and Martin 2012, 2016). Based on social contract, many legal writers suggest that any amendment to existing laws and passing of new law to protect privacy should take into consideration of the fact that sharing of information is part of individual autonomy rather than relinquishing of the right to privacy. The individual who shared the information should be allowed to retain the control of their information. As stated by the Privacy Commissioners in Canada, the images available in social media or any public domain should not have been uploaded without consent and would not have been expected to be used for mass surveillance. Additionally, the images and any other information in social media should be modified by the individuals concerned only and the individuals should exercise control over accessibility of content over time. The decision of the Canadian Privacy Commissioners is a catalyst, and such an approach is welcomed by many. However, we may not expect such decision from the judiciary in the absence of appropriate law. In the absence of legislation to address the use of invasive technology like FRT, firms and industries may follow ethics to self-regulate so that they can improve on consumer confidence. However, self regulation is often insufficient (Lochner, 2013; Selvadurai, 2015).

## 5 Conclusion

The FRT is being used by government entities for social welfare, defence, and law enforcement while the corporation is using it for understanding the customers and their preferences. FRT is an excellent tool that could be used for various purposes in a fast and cost-effective manner. Nonetheless, the use of FRT should be in line with the laws and ethical norms. Many a time right to privacy could be violated when the FRT is used for government and commercial purposes without consent or legally justifiable purposes. In many instances, the users either have no consent to use the data or exceeded the initial consent given for the collection and use. In such cases, the individuals whose privacy is violated do not have appropriate laws to stop the uses of their data and claim compensation for violation of their right.

Hence, the necessary amendment to the current telecommunication and criminal law to criminalise the unauthorised use of public images could deter violation of privacy. The uploading or storing or communicating the image of the non-consenting individuals and/or the identity of the individuals can be part of the criminal action (Lipton, 2009–2010). The intention of the party in uploading or storing or communicating the image through the unauthorized application of FRT could be considered as an element of the criminal action too. The law should also consider exceptions where use of FRT could be allowed without consent (Selvadurai, 2015).

In cases of application of common law principles to remedy privacy violation, there is a possibility to apply the existing principles like intrusion upon seclusion, breach of confidentiality, misrepresentation of commercial valuable name. However, they have limited application to novel circumstances created by technology like FRT. Further, the success of the application of the common law principles also depends on the willingness of the court to interpret liberally to extend the application of the traditional principles to new technology and phenomenon. An appropriate legislation in almost all the common law countries will clarify the criteria to use the FRT and related technologies, the limit of use and the exceptions. Such a legislative initiative could be a guideline for stakeholders who have interest in using FRT and other similar technologies.

Additionally, a comprehensive data protection law could add value in creating a balance between private and public interest for data. The data protection law could include provisions as to the conditions for data collection, data protection principles like maintaining accuracy, transparency, and the necessity to rely on current date. The requirement for human intervention in decision making on data that are generated using FRT should be mandated. The law should incorporate rights of the data subjects too. As FRT uses sensitive data that have lifelong effect, the requirement for approval before the use of FRT by multi-stakeholder will allow to get the view of various interested parties in the use of FRT. Introducing a regulatory oversight and safe keeping requirement of data could add sense of security as well as comfort. Any amendment to law or introduction of new law should acknowledge the fact that there is reasonable expectation of privacy when the images available in social media or any public domain as the images and any other information in social media can be modified by the individuals concerned and they exercise control over accessibility of content over time.

## Declarations

### Author contribution statement

Jawahitha Sarabdeen: Conceived and designed the experiments; Performed the experiments; Analyzed and interpreted the data; Contributed reagents, materials, analysis tools or data; Wrote the paper.

### Funding statement

This work was supported by 10.13039/501100012639Prince Sultan University.

### Data availability statement

No data was used for the research described in the article.

### Declaration of interests statement

The authors declare no conflict of interest.

### Additional information

No additional information is available for this paper.
